# Potential epigenetic mechanisms in psychotherapy: a pilot study on DNA methylation and mentalization change in borderline personality disorder

**DOI:** 10.3389/fnhum.2022.955005

**Published:** 2022-09-12

**Authors:** Yamil Quevedo, Linda Booij, Luisa Herrera, Cristobal Hernández, Juan Pablo Jiménez

**Affiliations:** ^1^Departamento de Psiquiatría y Salud Mental Oriente, Universidad de Chile, Santiago, Chile; ^2^Millenium Institute for Depression and Personality Research, Santiago, Chile; ^3^Department of Psychology, Concordia University, Montreal, QC, Canada; ^4^Sainte-Justine Hospital Research Centre, Montreal, QC, Canada; ^5^Programa de Genética Humana, Instituto de Ciencias Biomédicas, Universidad de Chile, Santiago, Chile; ^6^Escuela de Psicología, Universidad Adolfo Ibañez, Santiago, Chile

**Keywords:** borderline personality disorder, DNA methylation, mentalization, psychotherapy, epigenetics

## Abstract

Genetic and early environmental factors are interwoven in the etiology of Borderline Personality Disorder (BPD). Epigenetic mechanisms offer the molecular machinery to adapt to environmental conditions. There are gaps in the knowledge about how epigenetic mechanisms are involved in the effects of early affective environment, development of BPD, and psychotherapy response. We reviewed the available evidence of the effects of psychotherapy on changes in DNA methylation and conducted a pilot study in a sample of 11 female adolescents diagnosed with BPD, exploring for changes in peripheral DNA methylation of *FKBP5* gene, which encodes for a stress response protein, in relation to psychotherapy, on symptomatology and underlying psychological processes. For this purpose, measures of early trauma, borderline and depressive symptoms, psychotherapy outcome, mentalization, and emotional regulation were studied. A reduction in the average *FKBP5* methylation levels was observed over time. Additionally, the decrease in *FKBP5* methylation observed occurred only in those individuals who had early trauma and responded to psychotherapy. The results suggest an effect of psychotherapy on epigenetic mechanisms associated with the stress response. The finding that epigenetic changes were only observed in patients with early trauma suggests a specific molecular mechanism of recovery. The results should be taken with caution given the small sample size. Also, further research is needed to adjust for confounding factors and include endocrinological markers and therapeutic process variables.

## Introduction

Borderline Personality Disorder (BPD) is characterized by a general pattern of instability in affect regulation, impulse control, interpersonal relationships, and self-image. A revision of the data from The National Epidemiologic Survey on Alcohol and Related Conditions (NESARC) study in the United States estimates a lifetime prevalence of 2.7% (Trull et al., 2010). In mental health settings, the prevalence of BPD is expected to be 10% in outpatient and between 15% and 25% in inpatients (Leichsenring et al., 2011). The prevalence of BPD is relatively similar in adolescents and adults, and presents acute symptomatology, such as suicidal ideation, impulsive behaviors, and non-suicidal self-injury (NSSI; Stead et al., 2019). Adolescents with BPD have academic difficulties and social relationship problems (Kaess et al., 2017). Additionally, this disorder is a significant predictor of substance use and mood disorders (Chanen et al., 2008). Individuals with BPD symptoms at mean age of 14 years show lower life satisfaction, more general impairment, and need for services at mean age of 33 (Winograd et al., 2008).

Given the implications of BPD on the functioning and mental health of adolescents, a better understanding of the interplay between genetic and environmental factors that contribute to their symptomatic expression is highly needed.

The development of BPD pathology implies a complex etiopathogenic trajectory in which adverse early life events in the presence of genetic susceptibility that confer sensitivity to the environment, can lead to the development over time of the BPD phenotype or its underlying traits (Gunderson and Lyons-Ruth, 2008; Bulbena-Cabre et al., 2018). However, evidence of the underlying molecular mechanisms is largely unknown. A large-scale family study estimates the heritability of the disorder at 46% (95% CI = 39–53; Skoglund et al., 2021). Despite evidence from twin studies showing that BPD is highly heritable, genetic association studies are so far inconclusive (Calati et al., 2013; Amad et al., 2014).

Epigenetic processes are sensitive to environmental conditions and can operate as mechanisms that allow early environmental experiences to trigger phenotypic modifications without modifying the genotype (Weaver, 2007). A limited number of studies have shown relations between DNA methylation patterns and the presence of childhood stress in individuals with BPD symptoms, showing associations with genes involved in stress regulation and neuroplasticity. A positive correlation has been observed between the levels of DNA methylation of exon 1F the Glucocorticoid Receptor gene (NR3C1) promoting region, child maltreatment (physical abuse), and clinical severity in a sample of individuals with BPD (Martín-Blanco et al., 2014). Perroud et al. (2013) examined 115 individuals diagnosed with borderline personality disorder (BPD). All received intensive dialectical behavioral therapy. Those who responded to treatment showed a decrease in the percentage of exon I and IV *BDNF* DNA methylation. Changes in the methylation status were significantly related to changes in depressive symptoms, hopelessness, and impulsivity.

To date, there is no psychopharmacological treatment with robust evidence of effectiveness for the treatment of BPD. On the other hand, various types of specialized psychotherapy have shown efficacy in reducing symptoms and improving global functioning (Choi-Kain et al., 2017). These effects could occur by modification of the epigenetic profile. In line with the preceding, there are few recent studies relating BPD and the potential epigenetic effect of psychotherapeutic treatments, specifically on *BDNF* gene DNA methylation (Perroud et al., 2013; Thomas et al., 2018) and *APBA3* and *MCF2* genes DNA methylation (Knoblich et al., 2018). However, the exploration of the association between the change produced at the level of symptoms or personality functioning induced by psychotherapeutic interventions and changes at the molecular level is still very scarce (Jiménez et al., 2018).

Accordingly, the aim of the present study is to discuss the role of epigenetic changes as a mechanism of gene-environment interaction, its relevance for BPD, and current evidence on DNA methylation changes in individuals with BPD during psychotherapy. Second, the concept of mentalization as a capacity for the processing of the interpersonal context, its development from the early experiences of care, and its participation as a possible common factor in psychotherapy in BPD is exposed. Finally, the notion that subjective processing of the social environment can act on the genetic expression as a mechanism of adaptation of the BPD phenotype is discussed.

## Epigenetic Processes as A Mechanism of Gene-Environment Interaction

Epigenetic modifications refer to stable alterations of potential gene expression during development and cell proliferation, that are held through cell divisions and do not alter the DNA sequence (Jaenisch and Bird, 2003). These correspond to heritable patterns of DNA methylation and hydroxymethylation, posttranslational histone modifications, and gene expression regulation by non-coding RNAs (Zannas et al., 2015). The combination of these changes determines a specific pattern of gene expression, which is highly dynamic and permeable to environmental influences. It is also heterogeneous in different organisms, tissues, and cell types, and changes according to the stages of development. Therefore, they correspond to a complex set of mechanisms of “phenotypic plasticity” in response to environmental demands (Ecker et al., 2018).

Experimental models have studied the impact of early adversity as a function of maternal care. For instance, in rats, the effect of maternal care behavior like licking, grooming (LG), and back arch-nursing (ABN) on behavior of the offspring and DNA methylation in the glucocorticoid receptor gene (*NR3C1*) have been reported (Lutz and Turecki, 2014). Increased DNA methylation in promoter regions of GR gene (*NR3C1*; hence more inactive chromatin and therefore lower transcription) in the hippocampus of adult rats reared by mothers with low levels of LG-ABN compared to offspring reared by mothers with high LG-ABN was observed. This lower GR expression was associated with less negative feedback in the HPA axis and higher reactivity to stress (Lutz and Turecki, 2014).

In rats exposed to early stress (separation of mother and calf), increased secretion of corticosterone and a persistent increase in Arginine Vasopressin (AVP) expression in neurons of the paraventricular nucleus of the Hippocampus are observed. AVP acts by enhancing the action of Corticotropin Releasing Hormone (CRH) under sustained stress situations. This increase is associated with hypomethylation in the regulatory region CGI3 of* AVP* gene and with altered behaviors of stress coping (Murgatroyd et al., 2009).

In humans, a number of studies have explored the relationship between early adverse environment and changes in methylation patterns, using candidate genes and epigenome-wide strategies.

Regarding studies linking candidate genes studies related to the stress response and adverse events in childhood, a significant correlation between the number of adverse events reported and one exon 1F DNA methylation site (cg17860381, located in exon 1F) of* NR3C1* gene was observed in lymphocytes of females who reported childhood adverse events, including physical, emotional and sexual abuse. Moreover, this pattern of adverse events and DNA methylation was correlated with borderline symptoms (Radtke et al., 2015).

A systematic review conducted by Turecki and Meaney (2016) regarding the effects of social environment on *NR3C1* gene methylation in humans showed that there was a consistent relationship (16 out of 17 reviewed studies) between early life adversity and increased exon 1F DNA methylation across different tissues (blood, saliva, buccal cells, and brain tissue). However, the results are inconsistent when exploring the association between exon 1F methylation and psychopathology including post-traumatic stress disorder and Depression. These results are also inconsistent when exploring methylation in other sites of exon 1 of the same gene in relation to early adversity, suggesting the need for further research to determine the permeability and stability of DNA methylation of each specific site.

Different types of early adversity including physical, emotional, sexual abuse, or psychosocial deprivation are associated with altered DNA methylation in specific sites of the epigenome. The epigenome-wide studies that include a greater number of genetic loci, to date, are scarce, the sample sizes are small, assess early adverse events with different methods (Yang et al., 2013; Cecil et al., 2016; Kumsta et al., 2016; Perna et al., 2020; and Merrill et al., 2021) and their findings, are still inconsistent among them and with candidate genes studies.

## Epigenetics Changes and Psychotherapy in Bpd

A limited number of studies have explored the effect of psychotherapy on epigenetic changes in BPD.

One study was performed on a sample of 115 outpatients diagnosed with BPD (and 52 healthy controls) and extracted DNA from blood leukocytes before and after 4 weeks of Intensive Dialectical Behavioral psychotherapy (DBT) to measure CpG methylation of exons I and IV of the *BDNF* gene. Patients who responded to DBT, exhibited a decrease in DNA methylation of *BDNF* gene exons I and IV, whereas no association was found between BPD diagnosis and methylation levels (Perroud et al., 2013). In another study performed on 44 patients with BPD and 44 matched controls, DNA methylation of *APBA3* and *MCF2* genes was measured from blood samples. *APBA 3* (neuronal adapter protein) is related to the production of β-amyloid, a component of amyloid plaques linked to Alzheimer’s disease and *MCF2* is a guanine nucleotide exchange factor, involved in neurite outgrowth that has been associated with schizophrenia and autism-spectrum disorders. Individuals with BPD who respond to DBT therapy presented higher methylation in both genes after 12 weeks relative to non-responders (Knoblich et al., 2018).

A third study, involving a sample of 41 individuals with BPD and 41 healthy controls and assessing candidate gene DNA methylation, reported higher methylation levels in promoter IV of the *BDNF* gene in both saliva and blood samples of BPD patients. Twenty-six out of the 41 patients completed DBT psychotherapy and after 12 weeks, a decrease in methylation levels was observed only in saliva samples (Thomas et al., 2018).

Some results are discordant, for example, Perroud et al., (2013) study found significant DNA methylation change after therapy in blood samples while Thomas et al., (2018) work did not. One possible explanation may be that the first study reported an average of methylation from exon IV while the second reported individual CpG methylation. There were also differences in the methylation evaluation technique (high resolution melt analysis vs. pyrosequencing).

These initial findings suggest that psychotherapy may be associated with epigenetic changes in candidate genes related to neuroplasticity.

## *Fkbp5* Gene, Early Stress, Bpd, and Psychotherapy Response

*FKBP5* gene encodes for FK506, a glucocorticoid receptor co-chaperon whose levels are increased after stress exposure and decreases the ability of the glucocorticoid receptor to bind cortisol and to translocate to the nucleus, creating an ultrashort negative feedback for NR3C1 activation (Binder, 2009; Zannas and Binder, 2014). DNA methylation of FKBP5 gene promoting regions decrease gene transcription and might limit the effects over stress neuroendocrine response (Zannas and Binder, 2014).

An association has been described between a lower DNA methylation of intron 7 of the *FKBP5* gene and the presence of child maltreatment in adults (Klengel et al., 2013) and preschool children (Tyrka et al., 2015). Moreover, high FKBP5 DNA methylation was found in infants who displayed resistant attachment behavior (Mulder et al., 2017).

Changes in *FKBP5* DNA methylation have also been associated with other early stressors such as low socioeconomic status in childhood (Needham et al., 2015) and Holocaust survivors and their offspring (Yehuda et al., 2016).

These findings suggest a possible role for FKBP5 in the adaptation of molecular stress response systems in relation to the early environment through epigenetic modifications.

Interestingly, in a case-control study with individuals with BPD, *FKBP5* intron 7 (bin 2), DNA methylation inversely correlate with empathic perspective taking and with anxiety symptoms. Furthermore, lower empathic perspective-taking abilities and anxiety correlated with childhood maltreatment. Although this study does not find differences between clinical and non-clinical samples, it reveals the relationship between empathy engagement and *FKBP5* DNA methylation (Flasbeck and Brüne, 2021).

*FKBP5* DNA methylation has been also associated with response to psychotherapy in PTSD (Yehuda et al., 2013), children with anxiety disorders (Roberts et al., 2015), and agoraphobia (Roberts et al., 2019). Bishop et al. (2018) also report significant findings in individuals with PTSD treated with Mindfulness-Based Stress Reduction (MBSR) therapy, but in the opposite direction, with responders having increased DNA methylation (intron 7, bin 2).

## Mentalization in Borderline Personality Disorder

Mentalization can be understood as a mental activity that allows interpreting behavior in terms of intentional mental states of others (needs, desires, feelings, goals), constituting a form of social cognition (Fonagy and Luyten, 2009).

Through mentalization individuals realize that they have a mind that can mediate its experience with the world, have the capacity to distinguish the inner reality from external reality and include both, an intrapersonal mental context and emotional processes of interpersonal communication (Gergely et al., 2002).

The quality of early attachment relationships is critical for the development of mentalization, as they allow the internal states to be mirrored by an attentive and reliable caretaker. This process at the same time impacts the processes of emotional regulation and self-control (Fonagy and Luyten, 2009).

A mentalization deficiency occurs in subjects with BPD compared to a non-clinical sample and with subjects with other personality disorders, but only in the presence of child abuse (Fonagy et al., 1996). However, other studies show a superior capacity for mentalization in these patients. This may be because the expression of deficits could be in BPD, specifically activated in the context of attachment relationships under conditions of high emotional arousal (Antonsen et al., 2016), which increases the tendency to attribute mental states to others that exceed the information given by social cues (hypermentalization; Sharp and Fonagy, 2015).

Less certainty about mental states was reported measured by the Reflective Functioning Questionnaire (RFQ) in a sample of individuals with BPD compared to healthy controls (Morandotti et al., 2018). Moreover, individuals with BPD would not present difficulties in the processes of decoding mental states from observed behavior, but rather to make causal inferences and predictions that include context information and basic social knowledge (Németh et al., 2020). Lower values of mentalization ability measured with RFQ mediate the relationship between a diagnosis of BPD and insecure adult attachment, supporting the idea that the presence of negative internal work models reduces the ability to accurately distinguish the relationship between mental states and behavior (Badoud et al., 2018).

The treatment specifically developed to increase mentalization, Mentalization-based Treatment (MBT; Bateman and Fonagy, 2004) has shown to be effective compared to Structured Clinical Management in a sample of individuals with BPD in both reducing self-injurious behaviors and hospitalizations and in reducing symptoms and improving interpersonal functioning (Bateman and Fonagy, 2009). Also, at an 8-year follow-up, patients treated with MBT maintained a stable improvement over time compared with Treatment as Usual (TAU; Bateman and Fonagy, 2008).

Using the Psychotherapy Process Q-Set, an instrument that allows the rating of sessions according to how close they are to a prototypical session of their respective orientation, one study compared sessions of TFP, DBT, and therapy focused on mentalization. Interestingly, the prototype mentalization response correlated with all therapies, with a greater correlation on mentalizing the other (including the therapist) in TFP and more focused on the self in DBT (Goodman et al., 2015). These findings are in agreement with the statement that the development of mentalization corresponds to a common factor in BPD psychotherapies (Fonagy and Allison, 2014).

The capacity to navigate the interpersonal environment using the ability to mentalize can be improved by psychotherapies of different orientations in BPD. This change could be associated with biological changes at the epigenetic level specially in stress response systems.

## Pilot Study: Epigenetic Changes in Psychotherapy of Adolescents Diagnosed with Bpd

### Methods

This study aims to explore changes in peripheral DNA methylation of *FKBP5* gene, which encodes for a stress response protein, in relation to psychotherapy, symptomatology, and underlying psychological processes in a sample of 11 female adolescents diagnosed with BPD. For this purpose, measures of early trauma, borderline and depressive symptoms, psychotherapy outcome, mentalization, and emotion regulation were studied longitudinally at baseline, 3 and 6 months. Percentage DNA methylation levels of specific regions of *FKBP5* gene intron 7 were measured at the same time interval. The design was a quasi-experimental, longitudinal, process-outcome study.

### Participants

Participants were female adolescent patients, aged 15–20 years, with a BPD subthreshold cut-off of 3 or more criteria of the DSM IV-TR for BPD. Subthreshold-BPD was included based on impairment of quality of life, presence of self-injury, and suicidality similar to full-syndrome BPD female adolescents previously reported (Kaess et al., 2017). Participants were starting a psychotherapeutic process with a focus on difficulties in the development of their personality, eigth of them were of psychodynamic orientation and three were dialectical behavior therapy (DBT).

The exclusion criteria were the following: psychosis, pervasive developmental disorder, and unstable medical (non-psychiatric) disease.

### Recruitment

A convenience sampling technique was used by contacting psychotherapists who work with adolescent populations and whose theoretical model and therapeutic approach include the development of the mentalizing capacity, including psychodynamic psychotherapies and DBT applied in private practice and in public and private outpatient treatment centers of Santiago de Chile. Therapists were clinical psychologists or psychiatrists with formal therapeutic training. Verbal and written information about the research and subject participation were provided. The study was approved by the ethics committee of Pontificia Universidad Católica de Chile.

### Measures

#### Symptomatic profiling, process, and outcome questionnaires

Eligibility criteria was assessed through the Structured Clinical Interview for DSM-IV axis II Personality Disorders (SCID II; First et al., 1995) and Mini International Neuropsychiatric Interview (M.I.N.I-Kid; Sheehan et al., 2010). The presence of childhood trauma was evaluated using the Childhood Trauma Questionnaire (CTQ; Bernstein et al., 1997), while attachment patterns were evaluated usingthe Attachment Adolescent Questionnaire (AAQ; West et al., 1998). Once selected, the Brief Reflective Functioning Interview (BRFI; Rudden et al., 2005) and Difficulties in Emotion Regulation Scale (DERS; Gratz and Roemer, 2004) were applied at 0, 3, and 6 months. The Reflective Functioning Scale (RFS) coding system was applied to BRFI transcript by a certified coder (Fonagy et al., 1998). Also, as outcome measures the Youth Outcome Self-Report (Y-OQ-SR; Wells et al., 2003), Beck Depression Inventory (BDI-I; Beck et al., 1961), and Borderline Symptom List (BSL-23; Bohus et al., 2009) were applied at 0, 3 months and 6 months.

#### DNA methylation

In addition, a blood sample was collected to perform the epigenetic analysis at 0, 3, and 6 months. For each participant, a nurse collected three samples of 5 ml of venous blood. The genomic DNA of the participants was extracted from 5 ml leukocytes of peripheral venous blood using tubes with EDTA as an anticoagulant.

The methylation status of three intron 7 CpG sites (ADS3828-FS2, ADS3828-FS1, and ADS6607-FS) was determined individually as an artificial C/T SNP using QCpG software (Pyrosequencing Pyrosequencing method, Qiagen). The methylation level at each CpG site was calculated as the percentage of the methylated alleles divided by the sum of all methylated and unmethylated alleles. The mean methylation level was calculated using methylation levels of all measured CpG sites within the targeted region of each gene^1^.

### Data analysis

Data analysis was performed using R version 3.4.1 (R Core Team, 2016) and the R packages *psych* for descriptive data (Revelle, 2016). Baseline features were summarized using descriptive statistics. Given multiple points of data for each participant, a *mixed*-*effect growth curve modeling strategy was followed* to assess the change in time of DNA methylation and clinical parameters that account for shared variance within subjects while modeling between-subject differences using the nlme r package (Pinheiro et al., 2013).

Our model included average FKBP5 DNA methylation as the dependent variable, predicted by a linear function of time (i.e., Time = 0, 1, 2), dummy coded presence or absence of childhood trauma [based on at least one above-threshold score on the CTQ (Tyrka et al., 2015)], and genotype (where 1 means presence of T allele). Psychotherapy response was measured by the reliable change index (RCI) of the Y-OQ-SR. RCI was calculated using pre and post treatment score, standard deviation, and Cronbach alpha from the normative sample of the scale according to Jacobson and Truax formula (Jacobson and Truax, 1991; Bauer et al., 2004). The RCI allows to identify if the differences are greater than expected due to random error. An RCI that is greater than 1.96 correspond to the 97.5th percentile of a normal distribution and is equivalent to a statistically significant change (*p* < 0.05; Jacobson and Truax, 1991). The dependent variable was transformed to its natural logarithm to normalize the distribution of the residues. Visual inspections were performed to check assumptions of heteroscedasticity and normality of residues. Missing values (timepoints) were programmed to be omitted from each of the equations. Only random intercepts were accounted for, to avoid convergence problems due to the small sample size and number of parameters in the model.

## Results

The study was planned with 34 individuals based on a power calculation to detect a 10 percent difference in DNA methylation. Sampling was restricted by the onset of the COVID-19 pandemic. Two individuals dropped out of the study at baseline, and there were no significant differences in the number of symptoms. A total of 11 individuals completed the study. The mean age was 16.77 ± 1.64 years. Nine of the 11 patients fulfill the threshold of 5 BPD criteria and two patients fulfill the sub-threshold criteria with four symptoms. All patients were above the cut-off score for the presence of depression (BDI-I) of 13 for Chilean population (Valdés et al., 2017) and the mean was in the range of severe depressive symptomatology (Beck et al., 1988).

The presence of childhood trauma was determined if at least one of the subscales of the CTQ scored above the threshold for moderate trauma. Seven participants (63.6%) had scores in the “moderate to severe” trauma range on at least one of the subscales. No significant differences in DNA methylation were found at baseline between individuals with and without the presence of childhood trauma (0.79 vs. 0.78, *p* = 0.65).

Response to psychotherapy as measured by Y-OQ-SR and according to the Reliable Change Index was associated with a decrease in mean *FKBP5* DNA methylation only in those participants who reported the presence of moderate to severe childhood trauma (β = −3.18, SE = 1.24, *p* = 0.04; Table 1). Fixed effects explain 0.42 of the variance (marginal R^2^: 0.42, conditional R^2^: 0.81).

**Table 1:** Regression analyses of mean FKBP5 DNA methylation change in time according to trauma and psychotherapy response.

	β(SE)	*t*
Parameter
Fixed Effects
Intercept	75.03 (1.95)^**^	38.45
Time	−0.48 (0.68)	−0.70
Trauma	2.27 (2.22)	1.02
Psychotherapy Response	4.24 (2.55)	1.65
Genotype	1.73 (1.47)	1.17
Time × Trauma	−0.14 (0.89)	−0.02
Trauma × Psychotherapy Response	1.2 (0.96)	−0.45
Time × Trauma × Psychotherapy Response	−3.18 (1.24)^*^	−2.57
Random Effects
Intercept	2.12	
Residual	1.46	

Note: Estimates and standard errors (in parentheses). *p < 0.05, **p < 0.01.

No change was observed over time in levels of mentalization, nor was there any significant association with changes in DNA methylation.

No significant relationship was observed between genotype, depressive symptoms, borderline symptoms, emotional regulation, and change in DNA methylation over time.

## Discussion

In this study, a reduction in *FKBP5* DNA methylation was observed in responders to therapy and especially in the group with early trauma.

The finding of decreased *FKBP5* DNA methylation associated with response to psychotherapy replicates the results of previous studies in individuals with PTSD who were treated with exposure therapy (Yehuda et al., 2013), children with anxiety disorders treated with cognitive behavioral therapy (Roberts et al., 2015) and individuals with Agoraphobia with or without panic disorder (Roberts et al., 2019). Bishop et al. (2018) also report significant findings in individuals with PTSD treated with Mindfulness-Based Stress Reduction (MBSR) therapy, but in the opposite direction, responders have increased DNA methylation (intron 7, bin 2). This study found a decrease in *FKBP5* DNA methylation in BPD phenotype and with different types of psychotherapy (psychodynamic psychotherapy and dialectic behavior therapy) suggesting that psychotherapies, in general, can act as “environmental regulators” (Yehuda et al., 2013) through modification of expression of HPA-axis related genes across several mental disorders. DBT psychotherapy has previously been associated with DNA methylation change in other genes in individuals with BPD, but not with *FKBP5* (Perroud et al., 2013; Knoblich et al., 2018; Thomas et al., 2018).

The identification of plasticity genes can contribute to the advance in the identification of molecular markers of stable improvement in BPD. Interesting candidates are genes associated with the HPA axis, *NR3C1*, and *FKBP5*. Both showed stability in methylation for 2 years in healthy adults suggesting that it may be markers of stable changes and individual differences in stress response regulation (Di Sante et al., 2018). More research is needed to determine the patterns of variability and stability in time of methylation patterns of different genes, in different developmental periods and in clinical samples.

A striking finding of this study is that only those who reported the presence of early trauma exhibited a decrease in DNA methylation. This suggests that in this group the biological mechanism for developing BPD could be different than the no early trauma group.

Although in this study no difference in DNA methylation of FKBP5 intron 7 was found at baseline and no effect of FKBP5 SNP1360780 risk T allele, several studies have reported a relationship between the presence of childhood trauma and decreased DNA methylation levels in this region across different populations, preschool children, low-income adult population, Holocaust offspring, postpartum women, individuals with MDD, and individuals with psychotic disorders (Klengel et al., 2013; Yehuda et al., 2013; Tyrka et al., 2015; Tozzi et al., 2018; Grasso et al., 2020; Misiak et al., 2020) in particular those individuals carrying the *FKBP5* SNP1360780 risk T allele, suggesting the impact of early emotional environment on stress response systems and development of psychopathology throughout life.

No significant change in mentalization levels was observed, nor was an association found with response to psychotherapy or changes in DNA methylation. This is probably due to the difficulty of the instruments to detect changes in mentalizing capacity, which is highly context dependent. In individuals with BPD faced with interpersonal situations that generate emotional arousal, mentalizing capacity is deactivated and less sophisticated behavioral and emotional patterns are activated (Fonagy and Bateman, 2008). Instruments that can assess mentalization emerging from dyadic interaction such as psychotherapy sessions (Talia et al., 2019) could be more accurate and ecologically valid in finding episodes of mentalizing deactivations and mentalizing improvements across time.

In this study, only individuals who reported the presence of early trauma and who responded to psychotherapy exhibited a decrease in DNA methylation. Other studies have reported positive associations between the presence of early adverse events and response to psychotherapy, adult individuals treated for chronic depression responded better to psychotherapy than to psychopharmacological treatment if they had a history of childhood abuse (Nemeroff et al., 2003). Similarly, adolescents with non-suicidal self-injury (NSSI) had a better response to psychotherapy in reducing the frequency of NSSI if they had reported adverse childhood experiences (Edinger et al., 2020). This differential response to the presence of early trauma is suggestive of specific mechanisms not only of symptomatology development but also of distinct mechanisms of recovery. In this sample, the differential response to psychotherapy at the level of DNA methylation may imply that some individuals are more permeable at the molecular level to both negative (early trauma) and positive influences (psychotherapy) from their affective environment.

According to the differential sensitivity model, individuals carrying “plastic alleles” who, faced with an early sub-optimal affective environment, would be more susceptible to develop psychopathology but can be also more susceptible to respond to positive social environments (Hammen et al., 2015). Psychotherapeutic interventions, understood as a factor capable of modifying the relationship with the current social environment, may have a greater effect on individuals who carry plastic alleles (Leighton et al., 2017; Jiménez et al., 2018).

In accordance with the above, a GWAS study of twins reported a polygenic score based on differences in sensitivity to develop anxiety disorders according to positive or negative parenting. In a second sample, individuals with major differential sensitivity polygenic score responded better to individual cognitive behavioral therapy (Keers et al., 2016). These results suggest that those individuals who present a greater sensitivity to the environment present more emotional problems if they experienced negative parenting, but they will also be the ones who will benefit most from more intensive forms of psychotherapy (Choi-Kain et al., 2017).

Close human relationships regulate optimal stimulation and modulate arousal levels and attenuate stress in order to improve the adaptation to the social environment. This phenomenon has been called “psychobiological attunement”, and has been explored in mother-child dyads and peer relations and can be observed from its behavioral, physiological, and biochemical correlates (Field, 2012). For example, intrusive mothers can upregulate infants’ developing stress systems, increasing cortisol levels in saliva (Tarullo et al., 2017).

During the establishment of the therapeutic relationship, the formation of an alliance between patient and therapist can lead to the restoration of “epistemic trust”, that is, to restore an individual’s confidence in obtaining from another human being knowledge relevant to his or her adaptation to the social world (Fonagy and Allison, 2014). This would be particularly relevant with individuals with BPD, in whom insecure attachment patterns developed in sub-optimal interaction with their caregivers would involve chronic epistemic mistrust, i.e., a deficiency in the trustworthiness and relevance of interpersonal communication with the concomitant development of deficient behavioral and emotional patterns for establishing cooperation and the ability to repair ruptures in relationships with others, increase their mentalizing capacities and improve their adaptation to their social environment (Fonagy et al., 2015; Orme et al., 2019).

Psychotherapy as a special form of human relationship would then be constituted as a biologically embedded experience, capable of altering biological functions in a stable and long-term manner (Demetriou et al., 2015). Psychotherapy is constituted as a disrupter of the “external social recursion” that goes from the social environment to the neural systems, modifying the subjective perception of the interpersonal environment. At the same time, it is capable of changing the “internal physiologic recursion” that ranges from the Central Nervous System to gene expression, that includes hormonal systems, inflammatory molecules, and intracellular signal transduction (Slavich and Cole, 2013).

**Figure 1:**
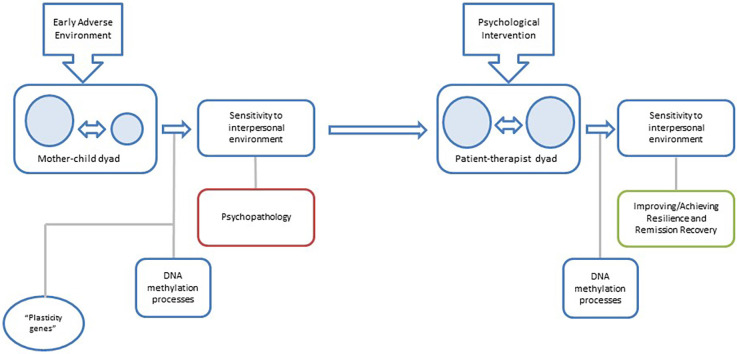
Proposal for a model of the relationship between changes in DNA methylation and psychotherapy in BPD: early mother-child interaction configures interpersonal sensitivity patterns on the child through DNA methylation processes, and the subsequent development of the borderline phenotype. In later stages of development, an appropriate patient-therapist interaction will be able to reconfigure interpersonal sensitivity patterns and reduce borderline symptomatology through stable changes in DNA methylation.

Psychotherapy focused on personality pathology may lead to changes in DNA methylation causing not only a symptomatic improvement but a reprogramming of the phenotypic adaptation to the interpersonal environment (Figure 1).

This study has several limitations; as a pilot study, our sample size was small relative to the number of predictors used in the final model, a known factor associated with type I and II errors. As such, our findings should be taken with caution and not be interpreted as conclusive but as a valuable indication of the direction for further inquiry in the study of the molecular changes associated with psychotherapy for personality problems, as they aim to inspire further studies with an adequate sample size. For the same reason, other concomitant environmental factors were not incorporated as covariates that may interact with DNA methylation such as physical factors, i.e., nutrition, alcohol, drugs, contraceptives, and sleep deprivation (Nilsson et al., 2016; Sarabi et al., 2017; Gabbianelli and Damiani, 2018) and other social factors such as socioeconomic status (Maddock et al., 2018) should be taken into account.

The absence of healthy controls is another important limitation because in the childhood and adolescent population there may be methylation changes associated with development.

In this regard, studies exploring longitudinal changes using a genome-wide DNA methylation strategy in adolescents show that in a range of 3–6 months, there is one group of genes that is highly variable over time and another that varies between individuals, but remains stable over time (Lévesque et al., 2014). Moreover, a study in 51 adult individuals showed stability of *FKBP5* DNA methylation for 2 years, suggesting that it could be a trait marker of stable changes and individual differences in stress response regulation (Di Sante et al., 2018). The present study was able to compare and found differences between responders and non-responders to psychotherapy i.e., those individuals who had no significant clinical change over time operate as controls, in a manner similar to other studies that longitudinally explored FKBP5DNA methylation changes in relation to psychotherapy (Yehuda et al., 2013; Bishop et al., 2018).

In this study, BPD symptomatology was assessed through an instrument based on symptom intensity according to the DSM-IV categorization (Bohus et al., 2009). The study of personality can be broadened by resorting to a dimensional approach in line with the Alternative Model of Personality Disorders of DSM-5 (American Psychiatric Association, 2013) and the International Classification of Diseases, 11th Revision [ICD-11, (ICD-11—Mortality and Morbidity Statistics, 2021)], for example characterizing the Functioning Levels of Personality, which include identity, self-direction, empathy, and intimacy (Zimmermann et al., 2012). These dimensions of personality functioning are more directly connected with the focus of the therapeutic work and, therefore, allow a greater understanding of the mechanisms of change in psychotherapy of personality.

Along with outcome indicators, psychotherapy process measures such as emotional regulation and mentalization were included which has not been reported in previous studies. Other process measures can be incorporated, such as therapeutic alliance (Horvath et al., 2011), characteristics of the therapist (e.g., warmth), and the patient (e.g., expectations; Wampold, 2015). Prospective, longitudinal studies designs with the use of repeated measures could allow exploration of the interaction between DNA methylation changes and different factors of the therapy process.

Differences in the direction of DNA methylation change may be due to different regions of FKBP5 having been explored, for example, the region near promoter exon 1 (Yang et al., 2021) or intron 7 different CpG sites (Roberts et al., 2019; Bishop et al., 2018). Potentially, a reduction in methylation could generate a downstream lower expression of the FK506-binding protein 5 and reduce glucocorticoid receptor resistance and the impairment of negative feedback loop (Yang et al., 2021), however, functional inferences are not possible given that FKBP5 expression and endocrine markers of hypothalamic-pituitary-adrenal axis function were not measured. Yehuda et al. (2013) found that variation in FKBP5 DNA methylation was associated with treatment response and correlated with measures of plasma cortisol and glucocorticoid sensitivity, implying a functional impact at the HPA axis level of changes in DNA methylation.

Despite the limitations, the present work proposes a design that allows us to explore the explanatory relationships between the therapeutic process and changes at the epigenetic level, in other words, to advance in the understanding of the molecular mechanisms of psychic change.

## Data Availability Statement

The original contributions presented in the study are included in the article, further inquiries can be directed to the corresponding author.

## Ethics Statement

The studies involving human participants were reviewed and approved by ethics committee of Pontificia Universidad Católica de Chile. Written informed consent to participate in this study was provided by the participants’ legal guardian/next of kin.

## Author Contributions

YQ, LB, LH, and JJ contributed to the conception and design of the study. CH contributed to data analysis. YQ wrote the first draft of the manuscript. All authors contributed to the article and approved the submitted version.

## Funding

This study was funded by The National Fund for Science and Technology (Grant N° 1150166), The National Agency of Research and Development (Grant N° 21150647), and by the Fund for Innovation and Competitiveness (FIC) of the Chilean Ministry of Economy, Development and Tourism, through the Millennium Scientific Initiative, Grant N° IS130005.

## Conflict of Interest

The authors declare that the research was conducted in the absence of any commercial or financial relationships that could be construed as a potential conflict of interest.

## Publisher’s Note

All claims expressed in this article are solely those of the authors and do not necessarily represent those of their affiliated organizations, or those of the publisher, the editors and the reviewers. Any product that may be evaluated in this article, or claim that may be made by its manufacturer, is not guaranteed or endorsed by the publisher.

## Footnotes

^1^ https://www.epigendx.com/d/service/pyrosequencing/dnamethylation
